# Effects of crossed states on photoluminescence excitation spectroscopy of InAs quantum dots

**DOI:** 10.1186/1556-276X-6-409

**Published:** 2011-06-02

**Authors:** Ching-I Shih, Chien-Hung Lin, Shin-Chin Lin, Ta-Chun Lin, Kien Wen Sun, Oleksandr (Alex) Voskoboynikov, Chien-Ping Lee, Yuen-Wuu Suen

**Affiliations:** 1 Department of Applied Chemistry, National Chiao Tung University, 1001 Da Hsueh Rd., Hsinchu, 30010 Taiwan; 2 Department of Electronics Engineering, National Chiao Tung University, 1001 Da Hsueh Rd., Hsinchu, 30010 Taiwan; 3 Institute of Nanoscience, National Chung Hsing University, 250 Kuo Kuang Rd., Taichung 402, Taiwan

## Abstract

In this report, the influence of the intrinsic transitions between bound-to-delocalized states (crossed states or quasicontinuous density of electron-hole states) on photoluminescence excitation (PLE) spectra of InAs quantum dots (QDs) was investigated. The InAs QDs were different in size, shape, and number of bound states. Results from the PLE spectroscopy at low temperature and under a high magnetic field (up to 14 T) were compared. Our findings show that the profile of the PLE resonances associated with the bound transitions disintegrated and broadened. This was attributed to the coupling of the localized QD excited states to the crossed states and scattering of longitudinal acoustical (LA) phonons. The degree of spectral linewidth broadening was larger for the excited state in smaller QDs because of the higher crossed joint density of states and scattering rate.

## Introduction

Self-assembled semiconductor nanostructures with three-dimensional carrier confinement provide the ultimate quantum system with discrete energy levels that can be tailored and controlled to tune the electrical and optical properties of these nanostructures. In particular, InAs on GaAs (001) self-assembled quantum structures is one of the most-studied systems. Quantum dots (QDs) provide another approach in making lasers, photodetectors, and memory devices as well as finding applications in quantum computing. Quantum dot lasers are predicted to have a high efficiency, low threshold current densities, and low temperature dependence of the threshold current [1-3]. The use of QDs may offer possibilities for low-power nonlinear devices. Therefore, the understanding of optical properties in these nanostructures is of extreme relevance for device applications to be a realistic prospect.

Theoretically, carriers confined in QDs show an atomic-like energy spectrum, characterized by discrete low-lying confined states, followed by spatially delocalized states associated to the InAs wetting layer and to the GaAs cap layer. The current perspective and analysis of the controversies regarding the phonon bottleneck in semiconductor QDs have been discussed by Prezhdo [4]. However, this simple picture fails when the actual dots are probed using advanced local probe techniques that provide excellent spatial and spectral resolutions. These techniques enable us to study only a few dots or even one dot. Near-field photoluminescence excitation (PLE) spectra of single quantum dots display 2D-like continuum states and a number of sharp lines between a large zero-absorption region and the 2D wetting layer edge [5]. The carriers were also found to relax easily within continuum states, and make transitions to the excitonic ground state by resonant emission of localized phonons. Limitations of the isolated artificial atom picture of an InAs QD were investigated in Ref. [6]. This study showed that the continuum background in the up-converted photoluminescence signal is possibly related to the wetting layer. Microphotoluminescence excitation spectra for neutral excitons revealed a continuum-like tail and a number of sharp resonances above the detection energy [7]. Temperature-dependent PL studies of an ensemble of self-assembled (In, Ga)As QDs provide insight into the nature of the continuous states between the wetting layer and QDs [8]. However, in contrast to other findings, PLE results of single InGaAs dot experiments by Hawrylak and co-workers showed spectra free of any continuum background and sharp emission lines [9].

Broadening of the QD bound states due to the influence of the couplings to the longitudinal optical phonons was discussed by Verzelen et al. [10], which revealed an effect of the dot environment to the dot eigenstates. Quantum kinetics of carrier relaxation in self-assembled QDs was investigated by taking into account the influence of the energetically nearby continuum of wetting layer states [11]. The interaction of the discrete QD excited states with a quasicontinuum of states was investigated in Ref. [12], in which a correlation between the acoustic phonon broadening efficiency and the background intensity in the PLE spectra of single InAs QD was found. The continuous background feature experimentally extends downward very deeply in energy, which makes it difficult to associate the fluctuations of the WL near the dot.

More recently, another source of intrinsic broadening for the dot bound states was demonstrated theoretically. This was stimulated during the interband optical excitation when electron-hole pairs are photo-generated in the dots, and is related to the existence of transitions involving one bound state and one delocalized state near the dots [13]. The broadening of the excited QD levels and interband absorption background are attributed to these cross transitions, which are inherent to the joint nature of the valence-to-conduction density of states in QDs.

This paper demonstrated that by engineering the QD size and shape, modification of the joint density of the cross transitions and changing the bound excited-state energies with respect to the continuum density of electron-hole states, which extend far below the wetting layer edge, can be made. The PLE spectroscopy of the self-assembled InAs QDs at low temperature and high magnetic field was reported. The resonances in the PLE spectra associated with the QD bound-state transitions were confirmed further by scanning the magnetic field. By evaluating the lineshape change of the PLE resonances, the coupling strength between the bound states and the cross transitions in different QDs was determined.

## Experimental details

**Figure 1:**
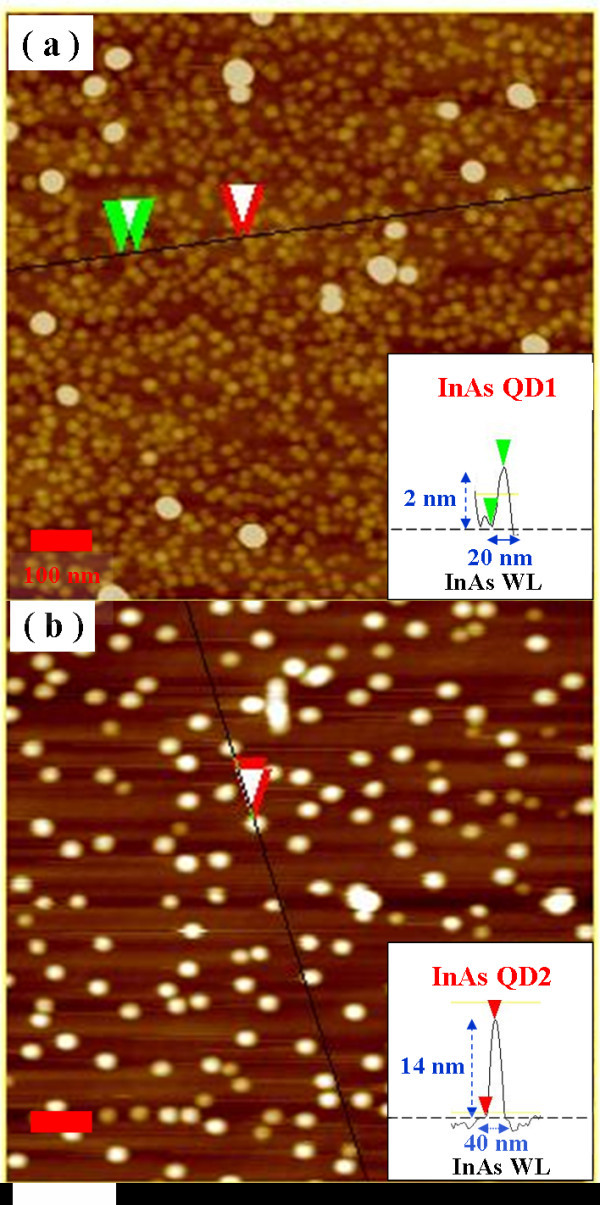
**Atomic force microscopy images of (a) QD1, (b) QD2**.

The two InAs QD samples studied in this work were grown on GaAs (001) substrates by molecular beam epitaxy. The substrate was first covered by a 200 nm GaAs buffer layer at 600°C. QDs of two different sizes were formed by depositing 2.4 and 2.6 monolayers of InAs with a growth rate of 0.056 μm/h at growth temperatures of 480°C and 520°C under As_2 _atmosphere, respectively. After the QDs were formed, they were then capped with a 150-nm thick GaAs Layer. QDs formed at a lower temperature (this sample is referred to as QD1 in this article) have a lens shape with a smaller average base diameter of about 20 nm and a height of 2 nm. The larger QDs (this sample is referred to as QD2 in this article) self-assembled at a higher temperature have a pyramid shape with an average base diameter of about 40 nm and a height of 14 nm. The areal densities are approximately 1 × 10^11 ^cm^-2 ^and 2 × 10^10 ^cm^-2 ^for QD1 and QD2, respectively. Figure 1a, b shows the AFM images of QD1 and QD2 samples.

The conventional PL spectra were obtained directly using an argon ion laser as the excitation source. PLE measurements for the above samples were recorded with a continuous wave (CW) tunable Ti:sapphire laser pumped with a DPSS laser as light source and detected using a 0.18-m double spectrometer equipped with a TE-cooled InGaAs photodetector. The samples were mounted in a closed-cycle helium dewar for low-temperature measurement. However, for the optical measurements under low temperature and high magnetic field, the sample was placed in a sample holder with N-grease at the bottom of an insert equipped with a fiber probe. The insert was placed in a dilution refrigerator and cooled down to 1.4 K. Laser output from the CW Ti:sapphire laser was delivered into the refrigerator using a fiber. The PLE signals collected through the same fiber were dispersed with a 0.55-m spectrometer and detected with a TE-cooled InGaAs photodetector at different magnetic field intensities.

## Results and discussion

**Figure 2:**
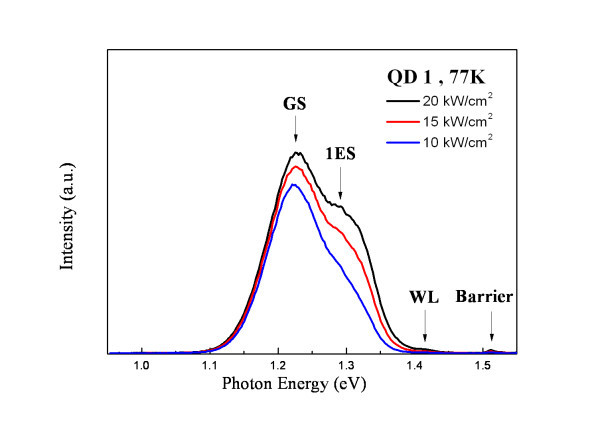
**Excitation power dependent PL spectra of QD1 at 77 K**.

**Figure 3:**
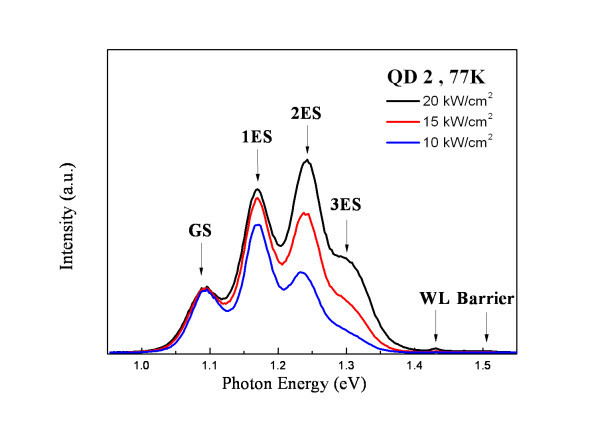
**Excitation power dependent PL spectra of QD2 at 77 K**.

Figures 2 and 3 show the excitation power dependence of the PL spectra at liquid nitrogen temperature for the QD1 and QD2 samples, respectively. The state filling of the excited state transitions can be observed for the QDs by increasing the pumping power. In Figure 2, the ground-state energy as well as the barrier and WL emissions of the QD1 are observed clearly at around 1.227, 1.509, and 1.422 eV, respectively. Due to the smaller size of the dots, only one excited state (1P_e _→ 1P_h _transition) was identified and contributed to the PL spectra at 70 meV above the ground state. For the dots with a larger diameter (QD2), there are three excited states contributed to the PL spectrum (as shown in Figure 3) at 86, 161, and 213 meV above the ground state other than the emission peaks from the barrier and the WL.

**Figure 4:**
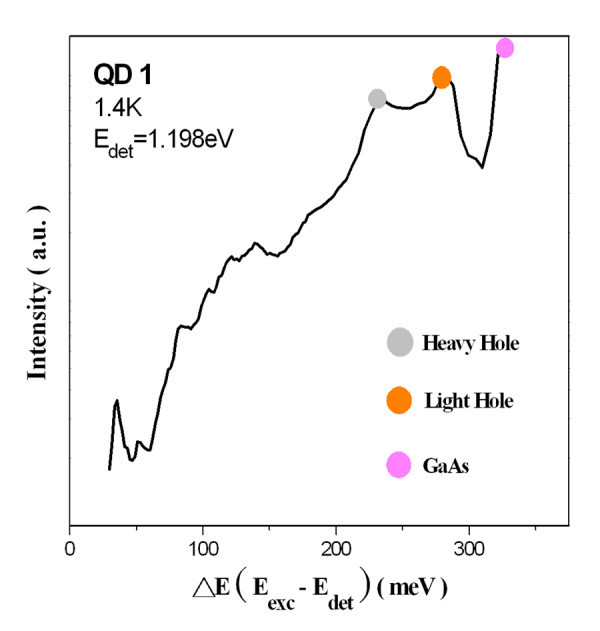
**PLE spectrum of QD1 was plotted as a function of relaxation energy recorded at 1.4 K**.

**Figure 5:**
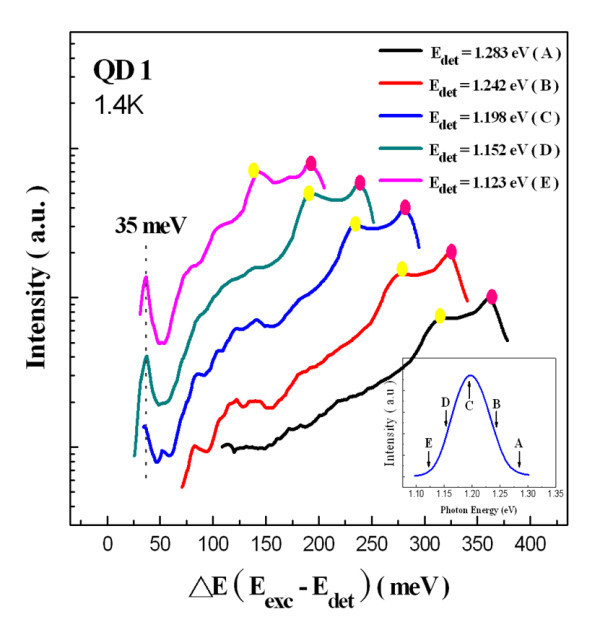
**PLE spectrum of QD1 recorded at 1.4 K with detection energy fixed**.

**Figure 6:**
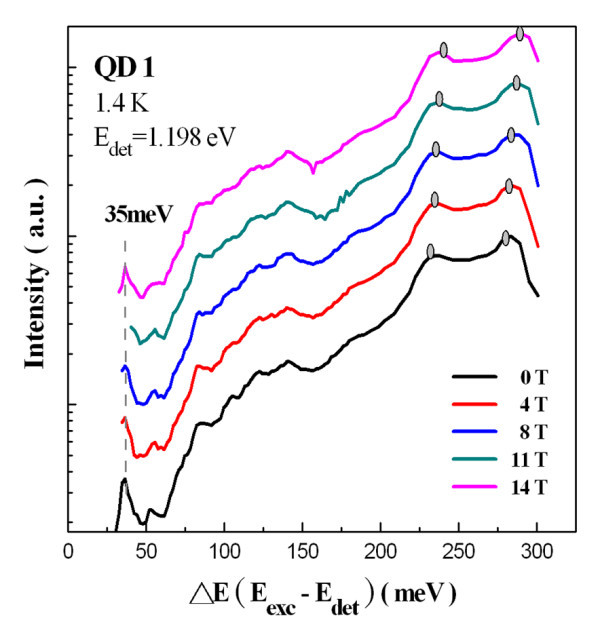
**Magnetic field dependent PLE spectra of QD1 recorded at 1.4 K from 0 to 14 T**.

For the PLE experiments, the excitation power was around 10 W/cm^2 ^to avoid any emission from an excited state and to suppress the Auger scattering. Figure 4 shows the PLE spectrum of the QD1 sample at 1.4 K with the detection energy fixed at 1.198 eV (the maximum of the ground state transition at the same temperature). In this figure, the spectrum with the bottom horizontal axis was plotted to represent the difference between the excitation and detection energies (*E*_exc _- *E*_det_). At the high-energy end of the PLE spectrum, absorption occurs in the GaAs barrier layer (approximately 1.52 eV) and in the 2D InAs wetting layer (absorption transitions from both the light holes and the heavy holes). At a lower energy, only one resonance at 35 meV above the ground state was observed. However, there was no peak resolved at the energy where the first excited state absorption happens. Figure 5 shows the PLE spectra of QD1 measured with detection energies fixed at five different positions on the ground state peak. All the absorption peaks, other than 35 meV, were shifted according to the change in detection energies. The peak at 35 meV also did not change in position when the PLE spectra were measured at different magnetic fields, as shown in Figure 6. All of the evidence indicate that the peak at 35 meV indeed corresponds to the relaxation by the emission of one InAs/GaAs interface phonon [14].

**Figure 7:**
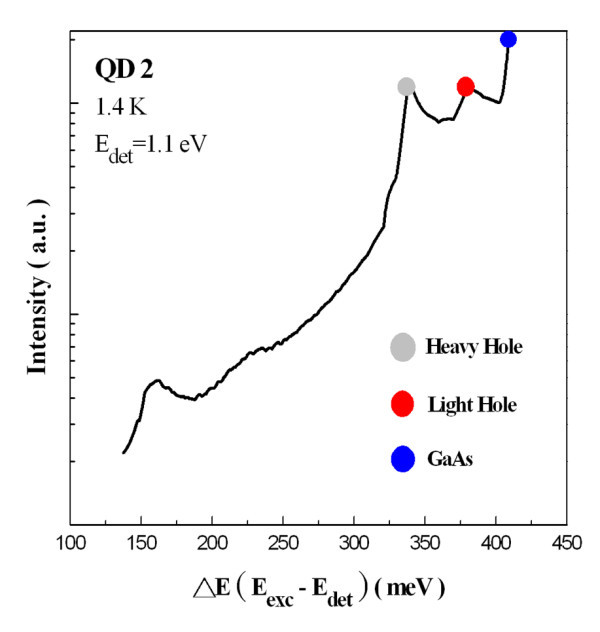
**PLE spectrum of QD2 was plotted as a function of relaxation energy recorded at 1.4 K**.

**Figure 8:**
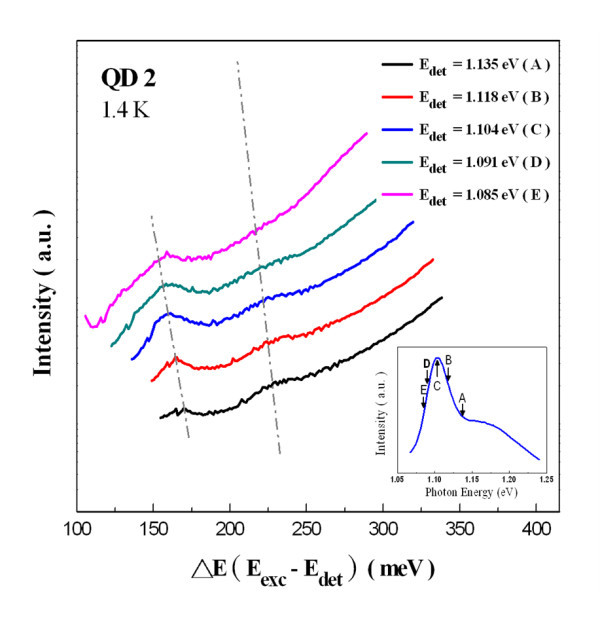
**PLE spectrum of QD2 recorded at 1.4 K with detection energy fixed**.

The PLE spectra of QD2 at 1.4 K are given in Figure 7 with the detection energy fixed at 1.1 eV (the maximum of the QD2 ground-state transition at 1.4 K). The energy of excited luminescence intensity was also displayed with respect to the detection energy (*E*_det_). Due to the limited laser tuning range, the PLE spectra down to 1.236 eV (i.e., 136 meV above *E*_det_) were recorded only. In contrast to the PLE results of smaller QDs, two resonance peaks were resolved clearly at 158 and 222 meV above the *E*_det_. However, the PLE resonance at 222 meV is much broader than the one at a lower energy. When PLE spectra recorded at five different detection energies (as shown in Figure 8) were compared, two PLE resonances clearly shifted according to the change in detection energy. Therefore, there is reason to believe that these two resonances are due to the bound-to-bound absorption.

**Figure 9:**
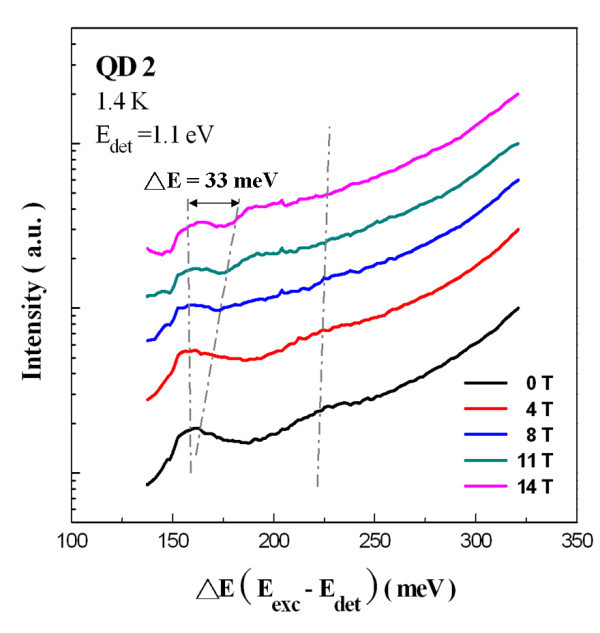
**Magnetic field-dependent PLE spectra of QD2 recorded at 1.4 K from *B *= 0 T to B = 14 T**.

To verify the resonances in the PLE signals, which are signatures from the QD excited-states transitions, PLE measurements under a magnetic field were performed. Figure 9 shows the magnetic field dependent PLE spectra of the larger QD sample with magnetic field scanned from 0 to 14 T. Splitting and a blue shift in energy were observed with increasing magnetic field for the two resonances at 158 and 222 meV, respectively. Under a magnetic field, the energy levels in 0D quantum structures can be modified by the effects of the diamagnetic shift or Zeeman splitting. The energy level modification due to the diamagnetic shift is relative small (approximately 1 to 2 meV) even at a magnetic field intensity of 14 T. The amount of energy splitting due to Zeeman effect, Δ*E*_Zeeman_, is given by the following equation: , where *m*_ℓ _is the angular quantum number of the 0D quantum structures, *e *is the electron charge, *ħ *is the Planck's constant, and *B *is the magnetic field. For excited state carrying angular momentum *m*_ℓ _of ± 1 (p-like state), the amount of energy splitting can be expressed as . By placing an effective mass of 0.05 m_0 _(for the InAs/GaAs quantum structures) into the above equation, a splitting in energy of about 32.39 meV was obtained for the p-like excited state transition. This number is quite close to the energy splitting (approximately 33 meV) of the resonance measured at 158 meV in our PLE spectrum. For the s-like transition, only the diamagnetic shift of approximately 1 meV is shown because *m*_ℓ _= 0. Therefore, the two peaks that appeared at 158 and 222 meV in the PLE spectra resulted from the transitions of p-like and s-like excited states, respectively.

**Figure 10:**
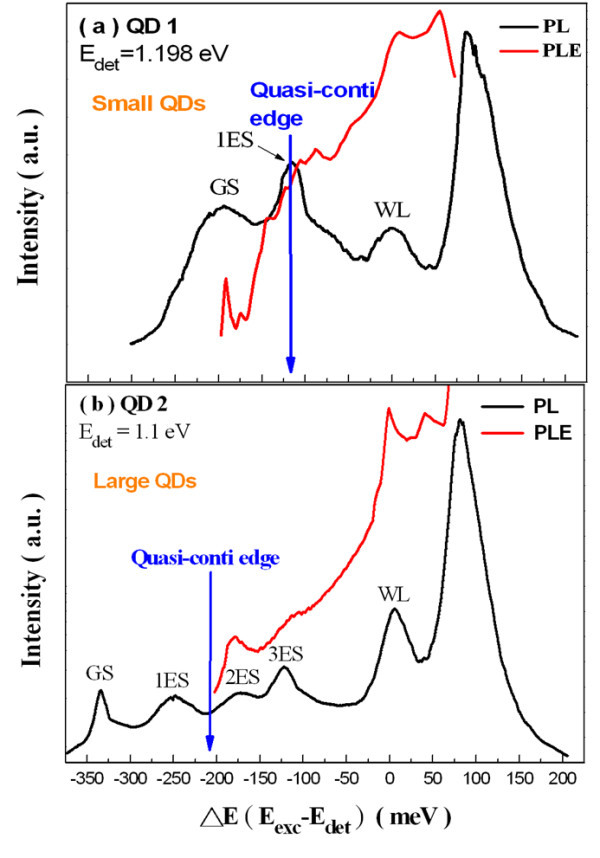
**PL and PLE spectra recorded at 1.4 K were plotted as a function of energy**.

In Figure 10a, b, the PL and PLE spectra from QD1 and QD2 at 1.4 K were displayed in parallel with the bottom horizontal axis representing the energy with respect to the WL absorption edge at 1.42 eV. As clearly shown in Figure 10b, the two emission peaks appeared in the PL spectrum of QD2 were still visible in the corresponding PLE spectrum with a slightly broadened resonance at approximately 125 meV measured from the WL. However, in contrast to the results from QD2, the emission peak of the first excited state at 115 meV measured from the WL in the PL spectrum of QD1 was missing in the corresponding PLE spectrum and was replaced by a random background, as shown in Figure 10a. The existence of a quasicontinuum of states has been suggested by the background signal observed in PLE and recently reported by different authors [5,15,16]. Recent experiments also revealed an efficient intradot relaxation mechanism which allowed the easy relaxation of the carriers within continuum states, and transition to the ground state by emission of localized phonons [5] or Auger scattering [17]. Quantitative study and analysis of the existence of acoustic phonon sidebands in the emission line of single InAs QDs was reported in Ref. [18]. The cross transitions provide a continuum of final electronic states that plays a role similar to the reservoir of large-wave-vector states for the excitonic ground state broadening in 2D quantum wells [12]. Therefore, the broadening of the excited state spectra lineshape and the interband absorption in QDs were affected by the interplay between cross transitions and LA phonon scattering.

The homogeneous linewidth Γ(T) of excitons is usually described as the sum of a static broadening *Γ*_0 _including radiative broadening and a temperature-dependent one, which accounts for acoustic and optical phonon broadening [19]. Efficient coupling to acoustic phonons exists not only for QD ground states but also for excited states. For low temperature, in which the interaction with acoustic phonon is dominant, linewidth broadening is written as [12,13]: *Γ*(*T*) = *Γ*_0 _+ *αT*, where *α *accounts for the acoustic phonon broadening efficiency. The acoustic phonon broadening efficiency *α*increased with the normalized background intensity [12].

**Figure 11:**
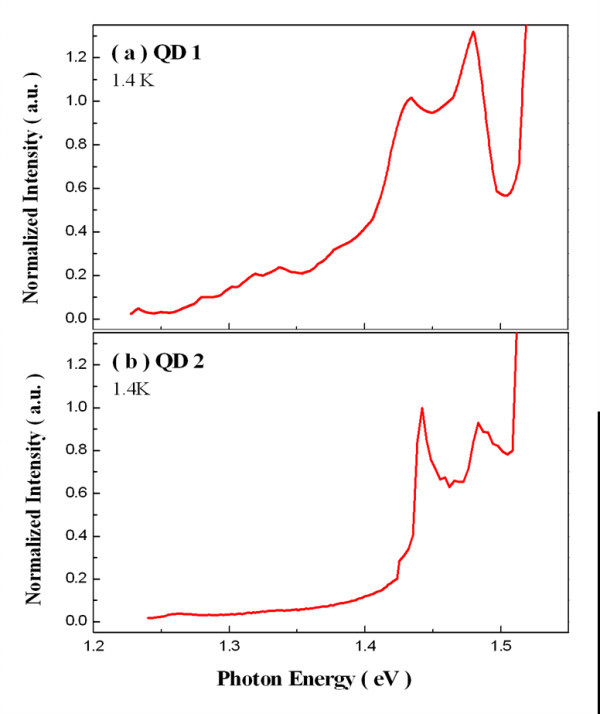
**Normalized PLE spectra with the PLE signal at 1.42 eV at 1.4 K**.

To interpret our experimental findings, bound-to-bound transitions and the bound-to-delocalized state transitions were calculated as a function of dot size at constant confinement potentials. A self-consistent iterative approach was used to calculate the energy levels of electrons and holes using non-parabolic and parabolic approximation for the conduction and valence bands, respectively. The calculations were done for a single dot in a large numerical box. The simulated dot geometries were determined from AFM measurements of our QD samples. The calculated onset energies of the continuum measured from the WL edge were 106 and 211 meV for QD1 and QD2, respectively, and are indicated with blue arrows, shown in Figure 10. Meanwhile, to compare different values of acoustic phonon broadening efficiencies *α *for QD1 and QD2, the QD-independent values of the PLE background signal needs to be determined. This was achieved by normalizing the PLE spectra with the WL absorption edge [12] at 1.42 eV for both QD1 and QD2, as shown in Figures 11a, b. However, the excited state of QD1 has the highest value in the normalized PLE intensity even though the energies of the excited states of both QD samples were above the onset energies of the continuum. Therefore, the excited state of QD1 had a higher acoustic phonon broadening efficiency and suffered more from the LA phonon-assisted scattering effect, which led to a much larger degree of linewidth broadening. Notably, the excited state of the QD2 at the higher energy also has a larger normalized PLE intensity than the excited state at the lower energy, which showed a broader spectral profile due to the higher phonon scattering rate.

## Conclusions

In conclusion, the magnetic field dependence of the PL and PLE spectra were measured for MBE-grown QD nanostructures with different sizes and shapes. The interband optical properties of QDs were affected by the crossed electron-hole levels. Additionally, The broadening of discrete (resonant) interband optical absorption is attributed to the combined effects of the crossed electron-hole levels and low energetic LA phonon scattering.

## Competing interests

The authors declare that they have no competing interests.

## Authors' contributions

CI carried out most of the experiments and calculations. CH and CP provided the QD samples. SC drafted the figures. Alex provided the software for carrying out the calculations. TC and YW helped with the low temperature-high magnetic field experiments. All authors read and approved the final manuscript.
